# Perceived Difficulty with Physical Tasks, Lifestyle, and Physical Performance in Obese Children

**DOI:** 10.1155/2014/735764

**Published:** 2014-07-06

**Authors:** Giuliana Valerio, Valeria Gallarato, Osvaldo D'Amico, Maura Sticco, Paola Tortorelli, Eugenio Zito, Rosa Nugnes, Enza Mozzillo, Adriana Franzese

**Affiliations:** ^1^Department of Movement Science and Wellness, University of Naples Parthenope, 80133 Naples, Italy; ^2^Department of Translational Medicine, University of Naples Federico II, 80131 Naples, Italy

## Abstract

We estimated perceived difficulty with physical tasks, lifestyle, and physical performance in 382 children and adolescents (163 obese, 54 overweight, and 165 normal-weight subjects) and the relationship between perceived physical difficulties and sports participation, sedentary behaviors, or physical performance. Perceived difficulty with physical tasks and lifestyle habits was assessed by interview using a structured questionnaire, while physical performance was assessed through the six-minute walking test (6MWT). Obese children had higher perceived difficulty with several activities of daily living, were less engaged in sports, and had lower physical performance than normal-weight or overweight children; on the contrary, they did not differ with regard to time spent in sedentary behaviors. Perceived difficulty in running and hopping negatively predicted sports participation (*P* < 0.05 and <0.01, resp.), while perceived difficulty in almost all physical activities negatively predicted the 6MWT, independently of BMI (*P* < 0.01). Our results indicate that perception of task's difficulty level may reflect an actual difficulty in obese children. These findings may have practical implications for approaching physical activity in obese children. Exploring both the perception of a task's difficulty level and physical performance may be useful to design exercise programs that allow safe and successful participation.

## 1. Introduction

Childhood obesity is one of the most serious public health challenges of the 21st century, since it presages adult obesity and is associated with the development of weight-related comorbid conditions and premature mortality. While the cardiovascular and metabolic consequences of pediatric obesity have been extensively studied [1], less attention has been paid to investigating the impact of obesity on physical functioning and disability in children. Physical inactivity in obese children may favor the development of a vicious circle perpetuating obesity, physical inactivity, and health risks [2]. Interrupting this concatenation of events is a central issue for managing weight loss. Most of the expert committees state that increasing physical activity levels and reducing sedentary behaviors, along with intensive dietary and cognitive-behavioral counseling, are the only key challenges and opportunities in the management of childhood obesity [3].

Even in children, obesity has a clear measurable negative impact on self-esteem, perceived athletic competence, physical appearance, and global self-worth [4]. It is important to consider the relationship among perceived difficulty with physical tasks, lifestyle, and physical performance in obese children as this evaluation may have important implications for clinical intervention to improve functioning, weight loss, and quality of life. Therefore, as primary research outcome we assessed perceived difficulty with physical tasks, lifestyle, and physical fitness in a clinical sample of obese children compared to normal-weight and overweight children. As secondary research outcome we explored the relationship between perceived difficulty with physical tasks and sports participation, sedentary behaviors, or physical performance.

## 2. Materials and Methods

This was a cross-sectional study in which 382 children and adolescents with chronological age range between 7 and 14 years participated. Obese children (*n* = 163) were consecutively recruited from the outpatient clinic of the Department of Translational Medical Science, University of Naples Federico II. The exclusion criterion was the presence of any specific genetic or endocrine pathologic process which may cause obesity. Controls were represented by a community sample of children (*n* = 240) recruited from an elementary school (3 classes) and a middle school (3 classes) in Naples by cluster sampling; from this sample 219 healthy children were considered (165 normal-weight and 54 overweight children), while obese children (*n* = 21) were excluded. The study started in January 2012 and ended in June 2013; it was approved by the review board of the Department. Written informed consent was obtained from all participants and/or their parents or legal guardians in accordance with the revised version of the Helsinki Declaration regarding research involving human subjects.

### 2.1. Anthropometric Measurements

Height and weight were measured with the children wearing only light clothes and no shoes; the BMI (weight (kg)/height (m^2^)) was calculated. Since height and BMI are age- and gender-related, these parameters were transformed into standard deviation score (SDS), based upon the established Italian BMI normative curves [5]. Overweight and obesity were defined according to BMI-SDS ≥1.04 and ≥1.64, which correspond, respectively, to the cut-off of 85th and 95th percentiles. Descriptive data are shown in Table 1.

### 2.2. Lifestyle

The study included a questionnaire assessment by interview regarding some behavioral issues of children, such as sports participation in the previous 6 months and sedentary habits. A sum of the daily hours spent in television viewing, videogames, and surfing on computer was computed to calculate time spent in sedentary behaviors.

### 2.3. Perceived Difficulty with Physical Tasks

Perceived difficulty with physical tasks was assessed by interview using a structured questionnaire, which included seven questions regarding physical limitations related to daily movement (walking, running, hopping, bending, stair climbing, feeling clumsy or awkward, and getting up from chairs), taken from the “mobility” domain of the Impact of Weight on Quality-of-Life adapted for adolescents [6]. Examples of questions included “How often do you have trouble with walking?” or “How often do you have trouble with using stairs?” or “How often do you feel clumsy or awkward?” For each mobility subscale, subjects had to select one among five possible responses ranging from “always” to “never.” Each answer was then rated on five-point scale ranging from 0 (never), 1 (rarely), 2 (sometimes), 3 (often), and 4 (always); a higher mobility subscale score indicates a greater level of impairment. A perceived global difficulty index was created by summing scores ≥2 for each physical limitation subscale.

### 2.4. Six-Minute Walking Test (6MWT)

The 6MWT was performed indoors along a flat, straight walkway in accordance with the American Thoracic Society guidelines [7]. The walking course length measured 20 m in the two different settings (hospital and school). The length of the corridor was marked every 3 m with a brightly colored tape. Cones were placed at either end of the walking course to indicate the beginning and end points. Additionally, the starting line, which marked the beginning and end of each lap, was marked on the floor using brightly colored tape. Instructions and demonstrations were given to each child. Participants were informed that the purpose of the test was to find out how far they could walk in 6 minutes and were instructed to walk the longest distance possible at their own pace during the allotted time. Hopping, skipping, running, and jumping were not allowed during the test. Only the standardized phrases for encouragement (e.g., “keep going,” “you are doing well”) and announcement of time remaining were given to the participants. Before and immediately following the test, the participant's hearth rate (HR) was recorded using a finger pulse oximeter (Smiths Medical PM, Inc., Waukesha, WI). Participants were tested individually in the presence of their parents or teacher. The 6MWT was administered by trained testers, who were expert in physical fitness assessment.

### 2.5. Statistical Analysis

Statistical analysis was carried out using the Statistical Package of Social Sciences (SPSS, Windows release 21.0; Chicago, IL, USA). Results are presented as mean ± standard deviation, with statistical significance set at *P* ≤ 0.05. The Kolmogorov-Smirnov goodness-of-fit test was applied for determining whether sample data likely derive from a normally distributed population. Variables not normally distributed were logarithmically transformed. However, for clarity of interpretation, results are expressed as untransformed values. The independent sample *t*-test or ANOVA was used to compare the means of continuous variables, while contingency table analyses were used for categorical variables. Pearson correlation coefficients, logistic regression, or linear regression analyses were performed to examine the relationship between perceived difficulty with physical tasks, sports participation, time spent in sedentary behaviors, and measures of 6MWT performance; age, gender, and BMI-SDS were included in the regression models. Clustering of active and/or sedentary behaviors was analyzed according to whether children reported any sports participation and/or ≥3 h/day spent in sedentary behaviors (this value represented the median hours spent in the whole group). Four categories were established, ranging from the most sedentary/least active group (≥3 h/day in sedentary behaviors and no sports participation) to the least sedentary/most active group (<3 h/day in sedentary behaviors and sports participation).

## 3. Results

### 3.1. Perceived Difficulty with Physical Tasks

Perceived difficulty with physical tasks was compared among normal-weight, overweight, and obese children. Perceived difficulty scores progressively increased from normal-weight to obese children (Table 2). Perceived difficulty scores in overweight children were significantly higher than normal-weight children in 4 out 7 tasks (walking, running, bending, and climbing stairs), whereas perceived difficulty scores in obese children were higher than both overweight and normal-weight children in all the explored tasks (*P* = 0.0001), except for bending (not significant versus overweight) or getting up from chairs (not significant versus normal-weight and overweight children, Table 2). Subjects who did not report any perceived difficulty with physical task were 72.1% in normal-weight, 46.3% in overweight, and only 18.4% in the obese group (*P* < 0.001 versus each other).

### 3.2. Lifestyle

Sports participation significantly differed among groups (obese 48.7%, overweight 63.0%, and normal-weight children 66.1%, *P* = 0.005); slightly more than half of the sample participated in individual sports, independent of the BMI-SDS category (obese 60.5%, overweight 52.9%, and normal-weight children 55.9%, *P* = 0.725). On the contrary, time spent in sedentary behaviors did not differ among groups; the percentage of children spending ≥3 hours/day in sedentary behaviors was 51.5% in obese, 48.1% in overweight, and 55.0% in normal-weight children (*P* = 0.647). Time spent in sedentary behaviors did not differ in children, whether or not they participated in sports activities (3.1 ± 2.2 versus 3.0 ± 2.2 hours, *P* = 0.772).

### 3.3. 6MWT

The distance achieved in the 6MWT (6MWD) was significantly lower in obese children than that covered by normal-weight and overweight children. Resting HR in obese children was higher than in normal-weight children (*P* = 0.05), while post-6MWT HR did not differ (Table 1).

**Figure 1:**
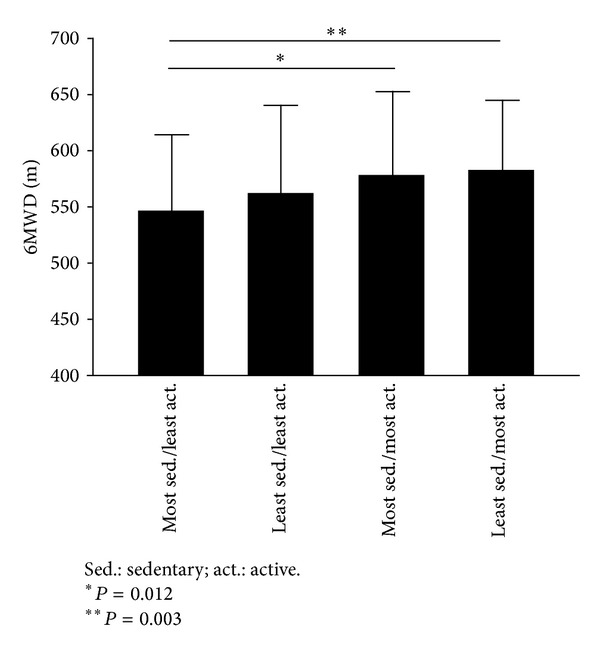
Distance achieved in the 6-minute walking test (6MWD) according to categories of children stratified for their lifestyle habits.

The 6MWD was compared among four different lifestyle categories, ranging from the most sedentary/least active group to the least sedentary/most active group. Children from the most sedentary/least active group covered the least distance with respect to the other groups (Figure 1). Interestingly, children with the most sedentary/least active lifestyle reported the highest perceived global difficulty index (1.8 ± 1.8) when compared with children of the other three groups (least sedentary/least active 1.5 ± 1.7, most sedentary/most active 1.1 ± 1.4, and least sedentary/most active 0.1 ± 1.4, *P* < 0.01). The percentage of obese children in the most sedentary/least active group was 48.8% versus 32.7% in the least sedentary/most active group (*P* = 0.013).

### 3.4. Relationships between Perceived Difficulty with Physical Tasks on Sports Participation, Sedentary Behaviors, and 6MWT

In order to analyze which factors may affect sports participation, logistic regression analysis including age, gender, BMI-SDS, and perceived difficulty with single physical tasks was performed in the total sample of children. Sports participation was negatively predicted by age, BMI-SDS, and perceived difficulty in running or hopping (Table 3).

Interestingly, perceived difficulty with physical tasks did not influence the amount of time spent in sedentary behaviors, except for “getting up from chairs,” which was independently correlated with sedentary behaviors, controlling for age, gender, and BMI-SDS (*B* 0.814, standardized beta 0.1, *P* = 0.05).

The 6MWD negatively correlated with perceived difficulty in physical tasks in the total sample (walking, *r*—0.330; running, *r*—0.344; hopping, *r*—0.282; climbing stairs, *r*—0.369; feeling clumsy or awkward, *r*—0.238; all *P* < 0.001; bending, *r*—0.116, *P* < 0.03). By linear regression analysis the 6MWD was positively associated with sports participation and negatively associated with BMI-SDS, and perceived difficulties in walking, running, hopping, climbing stairs, or feeling clumsy or awkward (*P* < 0.01), controlling for age and gender (Table 4).

## 4. Discussion

The main results of this study indicate that obese children had higher perceived difficulty with several activities of daily living, were less engaged in sports, and had lower physical performance than normal-weight or overweight children; on the contrary, they did not differ with regard to sedentary behaviors. Perceived difficulties in running and hopping negatively predicted sports participation, while perceived difficulties in almost all physical activities negatively predicted 6MWD, independently of BMI.

Engaging obese children and adolescents in physical activity requires addressing the individual, interpersonal, and environmental barriers that may deter them from participating in physical activities or sports [8]. Research on youth physical activity participation supports the strong influence of perceived competence and skill level on sports participation, especially in obese children [9, 10]. Among the individual barriers, perceived lack of physical competence is considered to be the most global construct of physical self-efficacy, representing people's overall perceptions of their general physical abilities on physical tasks. A review published by Tsiros et al. [11] indicated that greater weight was associated with lower health-related quality of life. More specifically, there was strong inverse relationship between physical functioning domain and BMI.

Our results demonstrate that perceived difficulty scores progressively increased from normal-weight to obese children and affected obese children in most of the daily physical skills, but at a lesser extent they also concerned overweight children. However, sports participation was significantly reduced only in obese children, and among those engaged in sport, most participated in individual activities. Indeed, being part of a team and having opportunities to demonstrate skills in front of friends and family may be particularly challenging and thus highly discouraging for obese children. Regarding sedentary habits, no difference was found indeed among obese, normal-weight, or overweight children confirming a previous research [12]. This finding is not surprising, since it has been reported that moderate-vigorous physical activity was independently associated with adiposity indices in children, while sedentary time was not [13]. As it has been suggested, physical activity and sedentary behaviors can coexist in the same individual [14].

To date only few studies have compared the 6MWT in overweight or obese children [15–17]. We found that obese children had lower functional capacity as measured by distance achieved in the 6MWT as compared to both normal-weight and overweight children, while no substantial difference was found between overweight and normal-weight peers, in agreement with other studies [15–17]. The 6MWT is a simple, practical, reliable, and valid measure of submaximal exercise capacity in children [18]. It is also considered as the most relevant walk test that reflects physical activity of daily living as well as cardiopulmonary fitness [19, 20] and its reproducibility has been demonstrated also in obese children and adolescents [15]. Similar to previous reports [16], we found that resting HR was higher in obese children than normal-weight or overweight children. Research has shown an increased sympathetic activity in obese individuals, including children that may thus explain this finding [21].

Perceived lack of physical competence is a subjective view of physical abilities that may or may not coincide with actual ability. Studies which have examined the effect of obesity on perceived physical impairment have failed to investigate whether impairments in body functions really translate into activity/participation restrictions [22]. The finding that perceived difficulties in almost all gait-related physical activities, such as walking, running, hopping, and climbing stairs negatively predicted the performance in the 6MWT may indicate that the estimation of difficulty level is likely to be accurate and may be used to assess the task's real difficulty. Remarkably, difficulties in bending or getting up from chairs had no effect, most likely because these activities are less involved with the gait task explored by the 6MWT. The observed changes in HR (after versus rest 6MWT) were fairly good and did not practically differ among normal-weight (+64%), overweight (+69%), and obese (+59%) children, indicating that motivation and attitude towards the test were the same in obese, normal-weight, or overweight children and that the 6MWT used in the current study was challenging enough to assess exercise capacity. It is interesting to note that perceived difficulty in getting up from chairs, which was the physical impairment less reported by children, significantly predicted time spent in sedentary behaviors, independent of BMI-SDS. Since this finding was unexpected, we did not test whether this perception translated into an actual difficulty. Further studies might explore whether this perceived difficulty is a real determinant or a consequence of sedentary habits.

Exercise performance was negatively affected by a lifestyle habit characterized by no sports participation and more time spent in sedentary habits, which involved more frequently obese children but was present also in normal-weight and overweight children. This result underlines that unhealthy lifestyle habits need to be tackled not only in children with excess weight, but also in normal-weight children, given the strong relationship between physical activity, physical fitness, and health [23].

A few limitations of our study can be acknowledged. For instance, our results cannot be extended to the whole population of obese children, since we specifically studied a clinical sample of obese children. It may be plausible that obese children who seek treatment may have worse health-related quality of life than obese children or adolescents who do not seek treatment [24]. In addition, overweight children were underrepresented; therefore some results may have been underestimated or undetected because of low statistical power. Lastly, our examination of physical performance was limited to walking test, while other tasks, such as those involving flexibility or strength were not assessed.

In summary, this study provides new data for clinical practice and adds to the recent research field on 6MWT performance in children who are overweight or obese. Based on theories of motivation and behavioral change, the best approach to increasing physical activity participation among obese children is to enhance their self-perception and enjoyment by increasing their actual and perceived motor skill competence. Therefore our findings may have practical implications for approaching physical activity in obese children. Exploring both the perception of a task's difficulty level and physical performance may be useful to discuss with obese children and their families the impact of excess weight on their daily physical activities. This information is essential to allow educators and trainers to design exercise programs that match the child's interests and physical abilities and to allow safe and successful participation in those activities which are less based on lower body loading, at least in the early steps of intervention.

## Figures and Tables

**Table 1:** Anthropometric data, lifestyle behaviors, and physical fitness of the study population.

	Normal-weight	Overweight	Obese
Number	165	54	163
Male/female	76/89	25/29	79/84
Age (years)	9.8 ± 1.7	9.6 ± 1.8	9.8 ± 2.3
Height (cm)	138.6 ± 10.8	142.2 ± 10.7	143.9 ± 13.1***
Weight (kg)	34.8 ± 8.8	46.3 ± 10.6	62.1 ± 18.3^§^
BMI-SDS	−0.17 ± 0.94	1.34 ± 0.17	2.28 ± 0.42^§^
Sports participation *n* (%)	109 (66.1)	34 (63.0)	79 (48.5)∗∗
Sedentary behaviors (h/day)	3.1 ± 2.2	3.0 ± 2.2	3.0 ± 2.1
6MWD (meter)	603.0 ± 67.1	591.2 ± 65.0	532.7 ± 61.1^#^
Resting HR (bpm)	86.2 ± 17.1	87.6 ± 18.6	90.6 ± 15.1*
Post-6WMT HR (bpm)	140.6 ± 29.9	148.9 ± 28.7	144.6 ± 25.5

*P = 0.05 obese versus normal-weight children; ∗∗*P* = 0.005 obese versus normal-weight and overweight children; ∗∗∗*P* < 0.001 obese versus normal-weight children; ^#^ *P* < 0.001 obese versus normal-weight and overweight children; ^§^ *P* < 0.001 obese versus normal-weight and overweight children; overweight versus normal-weight.

**Table 2:** Perceived difficulty with single physical tasks among normal-weight, overweight, and obese children.

	Normal-weight *n* = 165	Overweight *n* = 54	Obese *n* = 163	normal-weight versus overweight *P *	normal-weight versus obese *P *	overweight versus obese *P *
Walking	0.2 ± 0.7	0.5 ± 1.1	0.9 ± 1.2	0.014	0.000	0.025
Running	0.3 ± 0.7	0.7 ± 1.1	1.3 ± 1.3	0.001	0.000	0.001
Hopping	0.1 ± 0.5	0.3 ± 0.9	0.9 ± 1.3	0.200	0.000	0.001
Bending	0.1 ± 0.3	0.4 ± 0.9	0.6 ± 1.2	0.003	0.000	0.171
Climbing stairs	0.1 ± 0.5	0.5 ± 1.0	1.4 ± 1.4	0.014	0.000	0.000
Feeling clumsy or awkward	0.4 ± 0.7	0.6 ± 1.1	1.1 ± 1.3	0.353	0.000	0.008
Getting up from chairs	0.0 ± 0.3	0.0 ± 0	0.0 ± 0.3	0.410	0.163	0.154

**Table 3:** Binary logistic regression analyses predicting sports participation in the total sample of children.

	Independent variables	*B *	ES	*P *
Model 1	Age	−0.145	0.056	0.009
	Gender	0.015	0.221	0.945
	BMI-SDS	−0.281	0.090	0.002
	Walking	−0.055	0.108	0.612

Model 2	Age	−0.128	0.056	0.023
	Gender	−0.023	0.223	0.919
	BMI-SDS	−0.211	0.094	0.024
	Running	−0.219	0.107	0.041

Model 3	Age	−0.146	0.057	0.010
	Gender	−0.020	0.224	0.928
	BMI-SDS	−0.192	0.091	0.035
	Hopping	−0.363	0.117	0.002

Model 4	Age	−0.144	0.056	0.010
	Gender	0.011	0.221	0.959
	BMI-SDS	−0.272	0.088	0.002
	Bending	−0.128	0.122	0.296

Model 5	Age	−0.142	0.056	0.011
	Gender	0.024	0.221	0.914
	BMI-SDS	−0.265	0.095	0.005
	Climbing stairs	−0.067	0.099	0.496

Model 6	Age	−0.139	0.056	0.013
	Gender	0.025	0.221	0.910
	BMI-SDS	−0.277	0.090	0.002
	Feeling clumsy or awkward	−0.063	0.101	0.532

Model 7	Age	−0.144	0.056	0.010
	Gender	0.016	0.221	0.942
	BMI-SDS	−0.294	0.086	0.001
	Getting up from chairs	−0.182	0.411	0.658

**Table 4:** Linear regression analyses predicting 6MWD in the total sample of children.

	*R* ^2^	Adjusted *R* ^2^	Predictors	Standardized beta coefficients	*P*
Model 1	0.234	0.223	BMI-SDS	−0.341	0.000
			Sports participation	0.112	0.017
			Walking difficulty	−0.209	0.000

Model 2	0.223	0.212	BMI-SDS	−0.325	0.000
			Sports participation	0.099	0.038
			Running difficulty	−0.191	0.000

Model 3	0.208	0.197	BMI-SDS	−0.360	0.000
			Sports participation	0.097	0.046
			Hopping difficulty	−0.129	0.011

Model 4	0.195	0.184	BMI-SDS	−0.397	0.000
			Sports participation	0.118	0.015
			Bending difficulty	−0.022	0.654

Model 5	0.233	0.222	BMI-SDS	−0.307	0.000
			Sports participation	0.111	0.019
			Stair climbing difficulty	−0.220	0.000

Model 6	0.209	0.199	BMI-SDS	−0.362	0.000
			Sports participation	0.115	0.017
			Feeling clumsy	−0.131	0.009

Model 7	0.194	0.183	BMI-SDS	−0.403	0.000
			Sports participation	0.119	0.014
			Getting up from chairs	0.011	0.818
